# Microbial communities and metabolome profiles of fermented Chinese mustard greens from diverse regions in Guangdong province, China

**DOI:** 10.3389/fmicb.2026.1764488

**Published:** 2026-03-17

**Authors:** Sarengaowa, Jiayu Wang, Yongxi Kuang, Yun Ding, Hao Xie, Nan Diao, Siwei Shang, Man Hei Kam, Wenzhong Hu, Ke Feng

**Affiliations:** 1 College of Life Science, Zhuhai College of Science and Technology, Zhuhai, Guangdong, China; 2 Faculty of Medicine, Macau University of Science and Technology, Macao, China

**Keywords:** Chinese mustard greens, fermented vegetables, metabolite, metabolome, microbial communities

## Abstract

**Introduction:**

Fermented Chinese mustard greens (FCMG) is a well-known traditional fermented vegetable in Guangdong province, China. It has received considerable attention for the beneficial microorganisms and characteristic metabolite of FCMG. This study aimed to investigate the relationship between microbial communities and metabolome profiles of traditional FCMG from different regions.

**Methods:**

In this study, the microbial communities and metabolome profiles of traditional FCMG were evaluated by High-throughput sequencing (HTS) and metabolomics technology from households in Meizhou (MZ), Shaoguan (SG), and Zhongshan (ZS) in Guangdong province.

**Results:**

HTS analysis revealed *Lactobacillus* is the predominant microorganism in samples from three regions. The *Lactobacillus* abundance in ZS was significantly higher than that in MZ and SG. The relative abundance of Bacillus was found only in the MZ, whereas *Leuconostoc* and *Pediococcus* were only found in SG. The specific metabolite in sample identified was highest in MZ, followed by ZS and SG. In the overall analysis, the positive relationship was found between Lactobacillaceae and the accumulation of various organic acids (Cis-Sinapic acid, 5-Hydroxyferulic acid, 3-Feruloylquinic acid and 3- O-Caffeoyl-1-O-methylquinic acid). The positive relationship was also found between *Halomonas*, *Paenibacillus*, and volatile compounds (isothiocyanates, esters, terpenes, etc.).

**Conclusion:**

This investigation reveals the correlation of microbial communities and metabolite of FCMG from different regions. The finds provide scientific basis for screening benefit microbial which to develop high-quality flavor FCMG.

## 1 Introduction

The fermentation of vegetables in China can be traced back to 6,000 BC (Azam et al., 2017), and commonly used vegetables include mustard greens, cabbage, carrots, radishes, cauliflower, peppers, and bamboo shoots (Gunawardena et al., 2024). Fermented Chinese mustard greens (FCMG) have also become popular due to the beneficial microorganism, the characteristic secondary metabolites related with flavor and associated antioxidant activities. Mustard greens (*Brassica juncea* L.), is rich in vitamins, minerals, and dietary fiber, making it a broadly consumed and cultivated in Southwest, Central, East, and South China, China (Avato and Argentieri, 2015). Guangdong Province is known for its FCMG, due to its abundant mustard resources and excellent environment for the growth of fermentation microorganisms in China (Sarengaowa et al., 2024). FCMG, produced via spontaneous fermentation, are typically made in households in Guangdong Province, China. This process is carried out by the diverse natural microorganisms present on the surfaces of the Chinese mustard greens (Liu et al., 2021). Therefore, the quality and characteristics of FCMG are predominantly determined by their microbial communities, which play a critical role throughout the fermentation process.

Lactic acid bacteria (LAB) are the main microbial communities in fermented vegetables, including *Lactobacillus*, *Weissella, Leuconostoc*, and *Lactococcus* (Guan et al., 2022). LAB are widely recognized for conferring multiple health benefits such as enhancing immune function, competitively inhibiting harmful gut microbiota, and contributing to the management of serum cholesterol levels and blood pressure (Guo et al., 2024; Hashemi and Jafarpour, 2020; Yang S. et al., 2024). The dominant microbial communities of FCMG have a vital function in fermentation, directly influencing the metabolites composition and sensory quality of final product. Although the microbial communities of fermented vegetables have been increasingly evaluated, the specific functional microorganisms responsible for contributing to the characteristic metabolite of FCMG are rarely studied. Therefore, it is essential to evaluate the relationship between the dominant microbial communities and metabolites to achieve high sensory quality FCMG production.

The microbial communities during spontaneous fermentation are influenced by factors such as geographic position, climatic conditions, traditional fermentation methods, and environmental microbiota of raw materials cultivation, making consistent high-quality production difficult (Cao et al., 2017; Li et al., 2017). Therefore, understanding different regional microbial communities is essential, as they can affect the spectrum of metabolite changes during fermentation (Nie et al., 2017). Current research on FCMG includes the optimization of fermentation technology (Zhang et al., 2019), the screening of fermentation strains (Lin et al., 2020), the exploration of flavor formation mechanisms (Shen et al., 2018), and the improvement in quality and safety (Liao et al., 2019). However, there are still gaps in the knowledge on the microbial communities, metabolites and their functional associations in FCMG, particularly regarding samples from different regions.

Traditional FCMG are distributed in several main regions, including Meizhou (MZ), Shaoguan (SG), and Zhongshan (ZS), in Guangdong Province. It is necessary to research the differences in microbial communities and metabolites of FCMG at different regions. In this study, the microbial communities were analyzed by high-throughput sequencing (HTS) technology, and metabolites and different metabolites pathway was evaluated using metabolomics technology. Furthermore, the flavor quality correlation between the microbiota and metabolites was explored. This study could provide scientific basis for screening beneficial microbiota with the production of specific metabolites and developing high flavor quality FCMG products.

## 2 Materials and methods

### 2.1 Sample collection

This study selected three relatively far apart regions in Guangdong Province, China, namely Meizhou (MZ) (116°07′ E, 24°18′ N), Shaoguan (SG) (116°41′ E, 23°21′ N), and Zhongshan (ZS) (113°23′ E, 22°31′ N), which have significant geographical differences. In each region, we investigate and screen samples based on unified standards and select representative fermented mustard samples from households. These households have been fermenting Chinese mustard greens using traditional methods for over 15 years, and the flavor and quality of the FCMG is highly popular among the local community. Chinese mustard greens should be exposed to sunlight for 1 day to increase their degree of drying. The prepared mustard was placed in a fermentation tank. The traditional brine from household was added for fermentation purposes, accompanied by a small amount of salt. The container was sealed to initiate the fermentation process, which required a minimum duration of 15 days at the room temperature. The fermentation times of the MZ, SG, and ZS fermented mustard greens are 40, 15, and 30 days, respectively. Sampling was conducted as per the procedure mentioned by Yang et al. (2020). Samples were collected using sterile transfer pipes from five points each site (the top, middle, and bottom) at the same fermentation tank. The sample was mixed evenly and packed in sterile self-sealing bags. Six samples from each regions households (MZ, SG, and ZS region) were collected at the end stage of fermentation. Then, we placed the sterile self-sealing bags into a low-temperature sampling box containing ice packs and brought them back to the laboratory within 24 h. The samples were prepared for low-temperature storage for HTS and UHPLC-MS metabolome, with HTS testing in six parallel samples and UHPLC-MS metabolome testing in six parallel samples.

### 2.2 Microbial diversity sequencing of fermented Chinese mustard greens

#### 2.2.1 DNA isolation and PCR

Total microbial genomic DNA isolation was conducted from fermented Chinese mustard greens via the E.Z.N.A. R Soil DNA Kit (Omega Bio-tek, United States) as per the manufacturer’s guidelines. DNA integrity was tested by 1% agarose gel electrophoresis, and a NanoDrop 2000 spectrophotometer (Thermo Fisher Scientific, Wilmington, United States) was utilized to evaluate purity and concentration. Samples were preserved at −80°C until use. PCR amplification of samples and blank was conducted with an initial denaturation at 95°C for 3 min, then we conducted 27 cycles of 95°C for 30 s, 55°C for 30 s, and 72°C for 45 s, and a last extension at 72°C for 10 min. The purification of PCR outcomes was conducted via a PCR Clean-Up Kit (YuHua, China) and quantification was conducted with a Qubit 4.0 fluorometer (Thermo Fisher Scientific, United States) (Liu et al., 2016).

#### 2.2.2 Illumina sequencing and data processing

Pooling the purified amplicons was conducted in equimolar ratios and the Illumina NextSeq 2000 platform (United States) was utilized for sequencing as per Majorbio Bio-Pharm Technology Co., Ltd. (China) protocols. Raw FASTQ files’ demultiplexing was conducted with a custom Perl script, quality-filtered via fastp (v0.19.6), and assembled with FLASH (v1.2.7). We discarded reads with an average quality score < 20, length < 50 bp, or ambiguous bases. Sequences with ≥ 97% similarity were divided into operational taxonomic units (OTUs) via UPARSE (v11.0.667), and the most prevalent sequence was chosen as the representative. The classification confidence level is 0.7. Chloroplast sequences were manually removed, and taxonomic categorization was conducted via the RDP Classifier (v11.5) against the 16S rRNA gene.

### 2.3 Non-targeted metabolomics profiling

#### 2.3.1 Metabolite extraction

A 100 mg solid sample was positioned in a centrifuge tube (2 mL) with a grinding bead (6 mm) and isolated via 800 μL of methanol:water (4:1, v/v) comprising 4 internal standards (e.g., 0.02 mg/mL L-2-chlorophenylalanine). Grinding the samples was conducted for 6 min at −10°C and 50 Hz via a Wonbio-96 c frozen tissue grinder (Shanghai Wanbo Biotechnology Co., Ltd.), then ultrasonic isolation was conducted for 30 min at 5°C and 40 kHz. Samples were spun at 13,000 g for 15 min at 4°C after standing at −20°C for 30 min, and the collection of supernatant was conducted for LC-MS analysis.

#### 2.3.2 Quality control sample

Quality control samples (QC) are prepared as part of the system regulation and quality control process by mixing equal volumes of all samples. It helps to monitor the stability of the analysis, and the QC samples in this experiment were injected at the beginning, middle, and end of test.

#### 2.3.3 UHPLC-MS analysis

A UHPLC-Q Exactive HF-X system (Thermo Fisher Scientific) with an ACQUITY HSS T3 column (100 × 2.1 mm, 1.8 μm) (Waters, United States) was utilized to conduct liquid chromatography at Majorbio Bio-Pharm Technology Co., Ltd. (China). The mobile phase comprised 0.1% formic acid in water:acetonitrile (2:98, v/v) (solvent A) and 0.1% formic acid in acetonitrile (solvent B). The conditions were as follows: flow rate of 0.40 mL/min, column temperature of 40°C, and an injection volume of 5 μL. The UHPLC system was conjugated to a Q Exactive HF-X mass spectrometer with an electrospray ionization (ESI) source functioning in positive and negative modes. MS/MS analysis employed a normalized collision energy gradient of 20–40–60 V in rolling mode, and data were obtained via Data-Dependent Acquisition (DDA) with a mass range of 70–1,050 m/z.

#### 2.3.4 Metabolome profiles analysis

Progenesis QI (Waters, United States) was utilized to conduct LC/MS raw data preprocessing, generating a 3D data matrix exported in CSV format. Metabolite determination was conducted by querying the HMDB,^1^ Metlin,^2^ and an in-house MJDB (Majorbio Biotechnology Co., Ltd., China). Uploading data matrix to the Majorbio Cloud Platform^3^ was conducted for downstream analysis. Multivariate statistical analyses, such as principal component analysis (PCA) and orthogonal partial least squares-discriminant analysis (OPLS-DA), were conducted via the R ropls (v1.6.2). Metabolites with VIP > 1 and *p* < 0.05 were considered significance depending on variable importance in projection (VIP) from the OPLS-DA model and *p*-values from Student’s *t*-test. Mapping the differential metabolites between groups was conducted to biochemical pathways through enrichment analysis via the Kyoto Encyclopedia of Genes and Genomes (KEGG),^4^ and categorized as per their correlated pathways or biological functions.

### 2.4 Statistical analysis

The data were analyzed using SPSS software (version 14.0; SPSS, Chicago, IL, United States). The significance of differences between the variables was tested using one-way ANOVA. The means were compared using Duncan’s multiple range test. Statistical significance was determined at *p* < 0.05. Spearman’s correlation coefficient values were determined to access the correlation between microbiota and flavor metabolites using R 3.3.2. The *p-*values were adjusted by false discovery rate (FDR) using the Benjamini-Hochberg method, and only significant correlations (FDR < 0.05) were considered.

## 3 Results

### 3.1 Whole-system profiles of microbial communities from different regions

#### 3.1.1 Core microbial communities of fermented Chinese mustard greens

**FIGURE 1:**
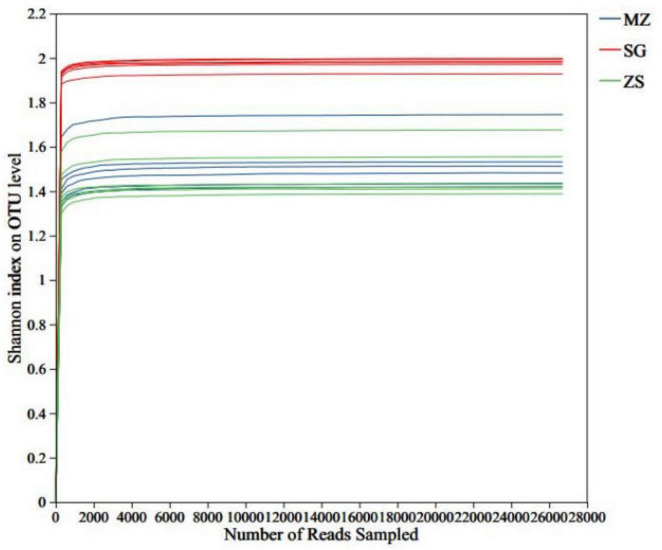
Sequencing dilution curves of fermented Chinese mustard greens from diverse areas of Guangdong Province, China. MZ, SG, and ZS represent Meizhou City, Shaoguan City, and Zhongshan City, respectively.

The sequencing the V3–h4 hypervariable region of the 16S rRNA gene was conducted, to detect the bacterial communities in FCMG from diverse regions. The number of high-quality sequences per sample ranged from 30,774 to 168,847, which were clustered into 61–145 OTUs (Table 1). The rarefaction curves approached saturation plateaus, and combined with high sequencing coverage, these outcomes illustrate that the sequencing depth was appropriate to capture most microbial diversity present. In addition, as shown in Figure 1, the trend curve tended to flatten, illustrating that the current number and quality of sequences were adequate to illustrate the majority of microbial populations in the FCMG samples.

**TABLE 1:** Number of sequences and values of alpha diversity index of fermented Chinese mustard greens from diverse areas of Guangdong Province, China.

Sample	Sequences (n)	Bases (n)	OTU (n)	Chao	Shannon index	Simpson index
MZ*1	38,409	16,464,831	135	146.50	1.42	0.30
MZ*2	32,660	13,997,832	130	148.75	1.48	0.30
MZ*3	34,085	14,596,059	145	158.15	1.74	0.32
MZ*4	135,957	58,255,583	137	158.08	1.53	0.35
MZ*5	118,843	50,926,864	130	143.80	1.44	0.39
MZ*6	130,181	55,780,355	136	140.3	1.51	0.36
SG*1	32,538	13,956,855	61	70.00	1.98	0.21
SG*2	128,446	55,096,285	66	75.43	1.98	0.19
SG*3	48,064	20,617,326	64	71.50	1.97	0.20
SG*4	154,590	66,313,580	64	82.33	1.99	0.19
SG*5	35,165	15,084,528	65	76.00	1.933	0.22
SG*6	168,847	72,429,186	62	73.00	2.00	0.20
ZS*1	40,767	17,473,844	103	111.00	1.43	0.35
ZS*2	38,538	16,521,812	92	100.57	1.39	0.35
ZS*3	51,773	22,191,436	94	109.30	1.42	0.35
ZS*4	30,774	13,187,462	95	97.77	1.68	0.28
ZS*5	76,397	32,731,022	110	156.20	1.56	0.33
ZS*6	36,252	15,537,919	100	115.00	1.41	0.37

*MZ, SG, and ZS represent Meizhou City, Shaoguan City, and Zhongshan City, respectively.

**FIGURE 2:**
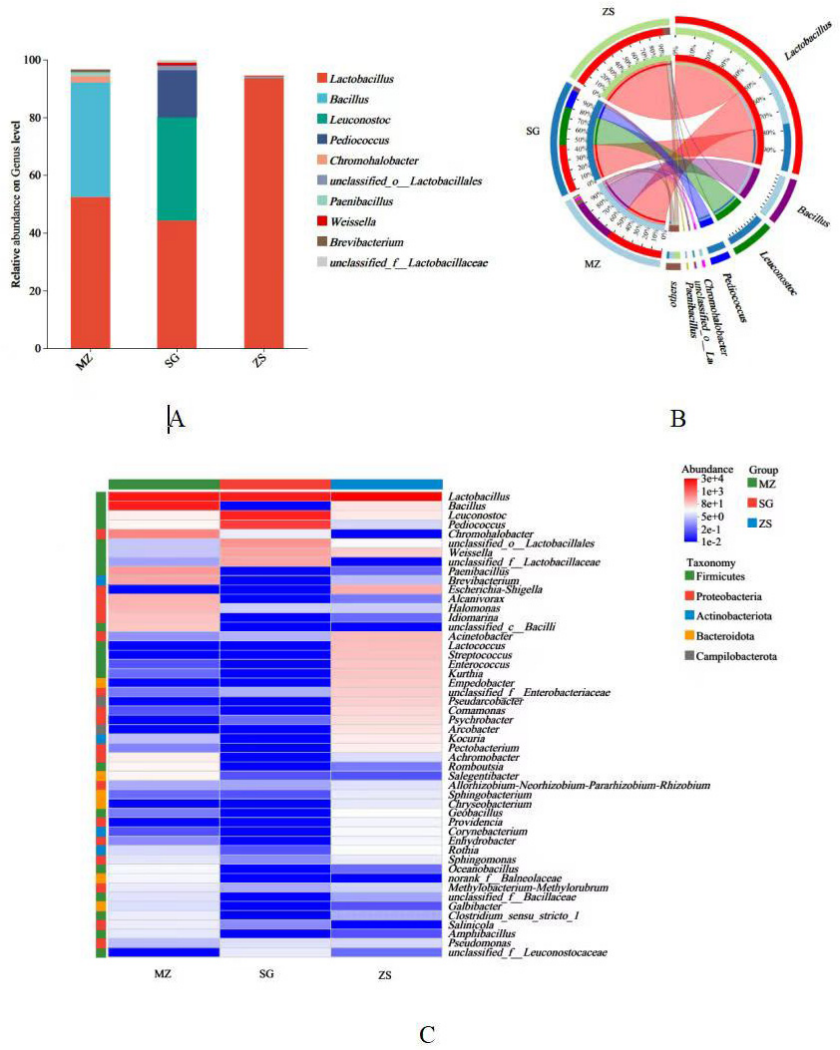
Relative abundance of microbial communities in fermented Chinese mustard greens from different regions of Guangdong Province, China. Bar plots **(A)**; Circos plots **(B)**, and Heatmap **(C)**. MZ, SG, and ZS represent Meizhou City, Shaoguan City, and Zhongshan City, respectively.

The microbial communities of FCMG were shown in Figure 2. At the bacterial genera level, the main genera in FCMG from three regions mainly include *Lactobacillus, Bacillus, Leuconostoc, Pediococcus, Chromohalobacter*, *Paenibacillus, Weissella*, and *Brevibacterium* (Figure 2A). Among these, *Lactobacillus* was prominently present in MZ, SG, and ZS, accounting for 52, 44, and 94% of the samples, respectively (Figure 2B). *Lactobacillus* has a vital function in the fermentation procedure, converting sugars into lactic acid through fermentation. This process not only imparts a unique flavor to fermented food but also reduces the pH value of FCMG, thereby inhibiting the growth of harmful microorganisms and extending the shelf life. Meanwhile, *Lactobacillus* can synthesize vitamins such as vitamin B and vitamin K during the fermentation process, thereby enhancing the nutritional value of the food.

Figure 2C illustrates the heatmap and sample clustering tree of the phylum-level species composition of FCMG. The heatmap displays 50 genera from five phyla, namely Firmicutes, Proteobacteria, Actinobacteriota, Bacteroidota, and Campilobacterota, with Firmicutes dominant. In addition, the heatmap and sample clustering tree also showed that the dominant genus among the MZ, SG, and ZS was *Lactobacillus*. The advantageous phylum compositions of MZ and SG are similar, but different from that of ZS.

#### 3.1.2 Alpha and beta diversity analysis

**FIGURE 3:**
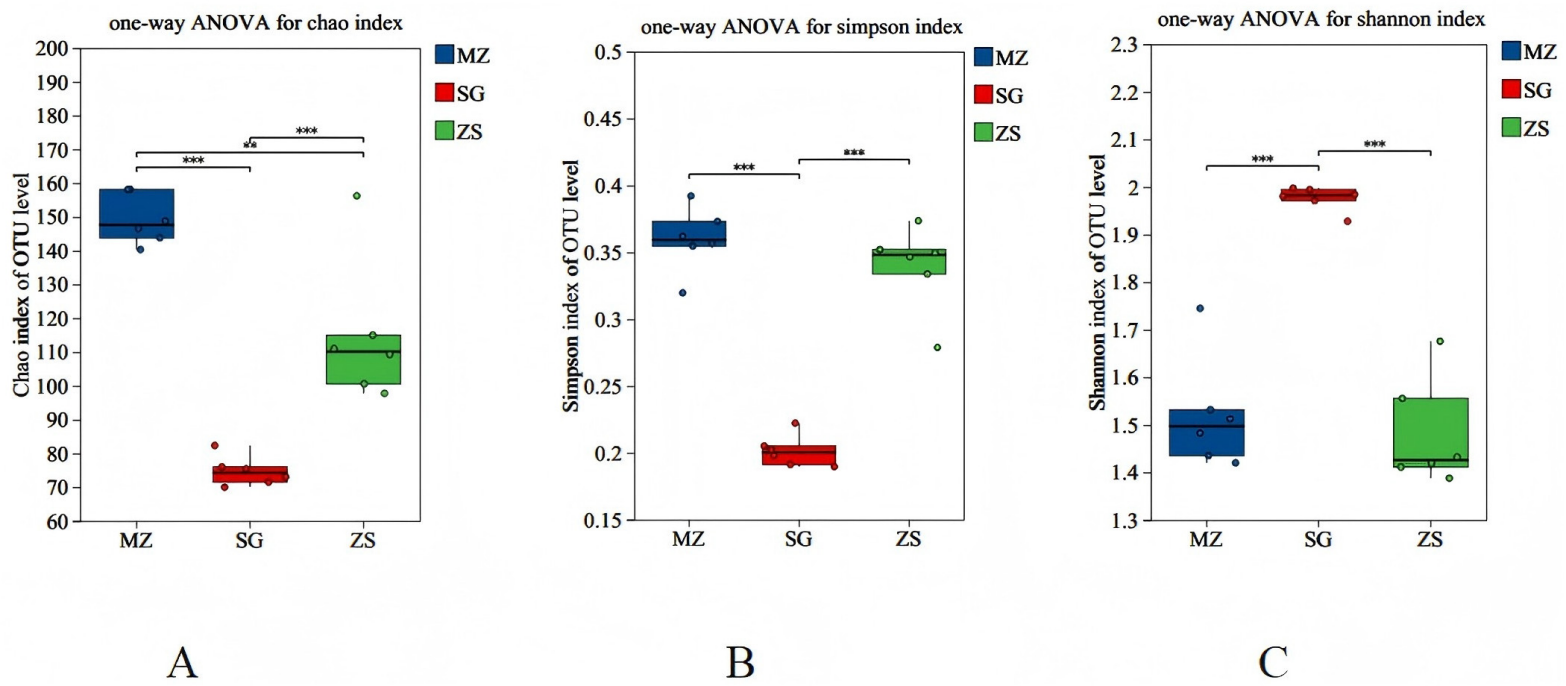
Alpha diversity of fermented Chinese mustard greens from diverse areas of Guangdong Province, China. **(A)** Chao index; **(B)** Simpson index; **(C)** Shannon index. MZ, SG, and ZS denote Meizhou, Shaoguan, and Zhongshan Cities, respectively. Boxplot center, upper, and lower lines represent the median and the first and third quartiles. ** and *** denote significance at *p* < 0.05, 0.01, and 0.001, respectively.

The alpha diversity captures the species richness, diversity, and consistency within locally homogenous surroundings. The box plots in Figure 3 offer a visual depiction of the differences in alpha diversity across multiple groups. The outcomes illustrated that there were significant differences in the detected OTU richness (Chao index) among the three sample groups, and the microbial species richness was ranked from high to low as MZ > ZS > SG, which were 149.27, 74.71, and 114.97, respectively (MZ vs. SG, *p* < 0.001; MZ vs. ZS, *p* < 0.01; SG vs. ZS, *p* < 0.001) (Figure 3A), which aligns with the outcomes revealed in Figure 2B. According to the Simpson indices of sample community diversity, there are significant differences in microbial species between MZ (0.36) and SG (0.20), SG (0.20) and ZS (0.34) (MZ vs. SG, *p* < 0.001; SG vs. ZS, *p* < 0.001) (Figure 3B). Moreover, according to the Shannon indices of sample community diversity, there are significant differences in microbial species between MZ (1.52) and SG (1.98) and SG (1.98) and ZS (1.48) (MZ vs. SG, *p* < 0.001; SG vs. ZS, *p* < 0.001) (Figure 3C).

**FIGURE 4:**
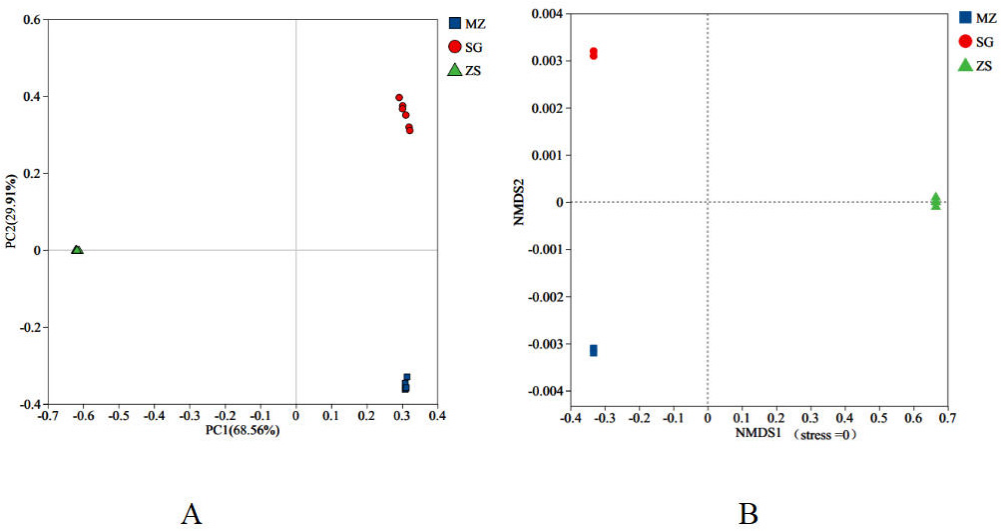
Beta diversity of fermented Chinese mustard greens from diverse areas of Guangdong Province, China. **(A)** Principal coordinates analysis. **(B)** Non-metric multidimensional scaling analysis. MZ, SG, and ZS represent Meizhou City, Shaoguan City, and Zhongshan City, respectively.

Beta diversity was evaluated via principal coordinates analysis (PCoA) and non-metric multidimensional scaling (NMDS). In PCoA, the contribution rate of the 1st principal component was 68.56%, the contribution rate of the 2nd principal component was 29.91%, and the total contribution rate was 98.47%, indicating that the model can correctly identify all samples of fermented Chinese mustard greens from the diverse areas. In NMDS, a stress value < 0.05 indicates good representativeness; herein, the stress value was 0, demonstrating excellent representativeness. Different colors or shapes denote sample groups, and closer points indicate greater similarity in species composition. As shown in Figure 4, microbial communities in fermented Chinese mustard greens from MZ, SG, and ZS regions were distinctly separated, indicating significant regional differences consistent with the alpha diversity results.

#### 3.1.3 Species differences and association network analysis

**FIGURE 5:**
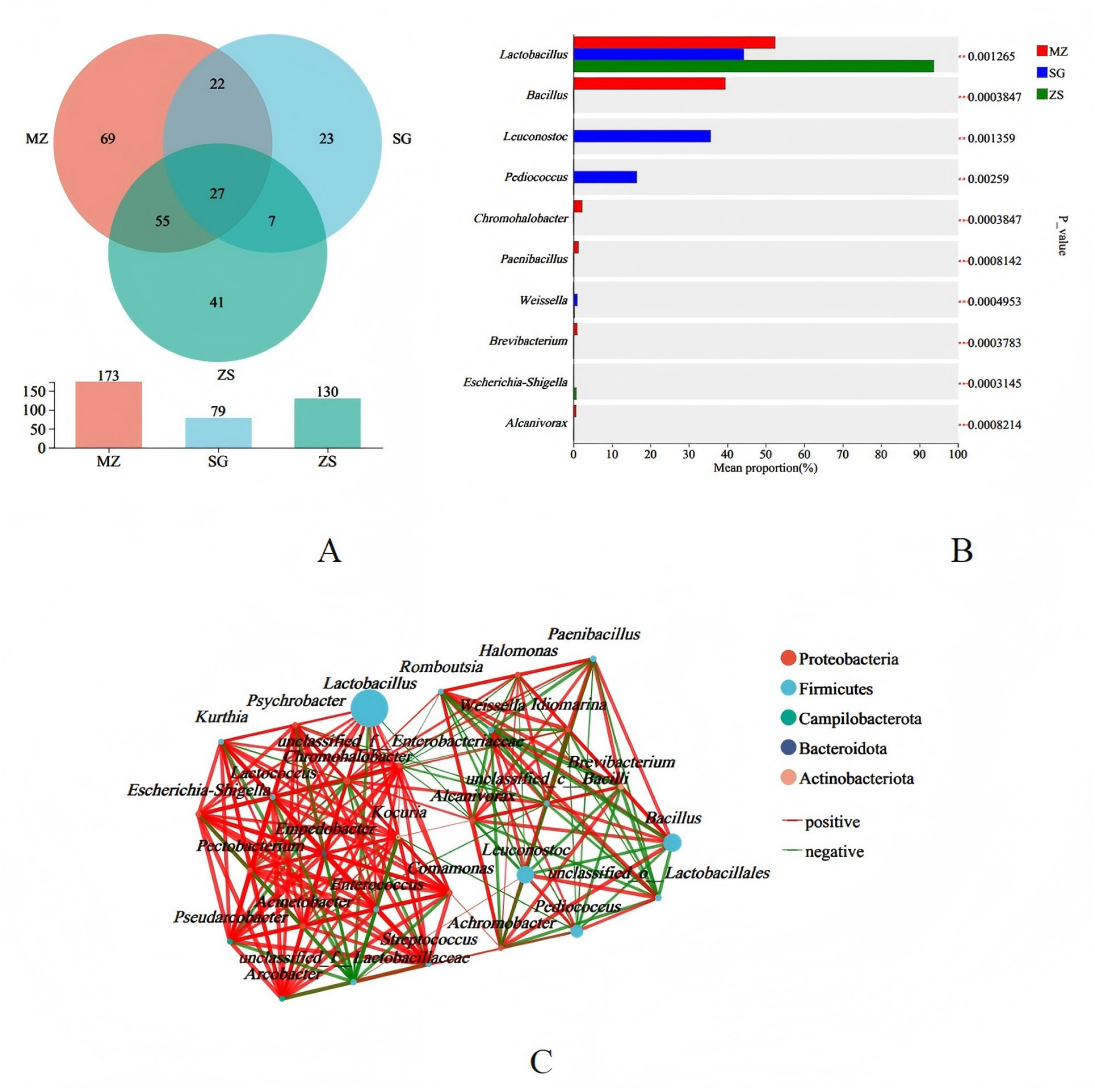
Species difference and association network analysis of fermented Chinese mustard greens from different regions of Guangdong Province, China. **(A)** Species Venn diagram. **(B)** Significant difference test between groups. **(C)** Association network analysis. MZ, SG, and ZS represent Meizhou City, Shaoguan City, and Zhongshan City, respectively.

Variations in microbial communities contribute to differences in flavor quality in FCMG from the MZ, SG, and ZS regions. Therefore, identifying these compositional differences is essential. Venn diagram analysis showed 27 shared species among the three regions, with region-specific species ranging from 23 to 69 (Figure 5A). Significant intergroup differences were observed for genera such as *Lactobacillus*, *Bacillus, Leuconostoc, Pediococcus*, *Chromohalobacter, Paenibacillus, Weissella, Brevibacterium, Escherichia-Shigella*, and *Alcanivorax* (*p* < 0.01 or *p* < 0.001) (Figure 5B). The microbial association network illustrated positive and negative correlations between taxa, with line thickness indicating correlation strength. As shown in Figure 5C, microbial communities in the fermented samples exhibited predominantly strong positive correlations.

#### 3.1.4 Prediction of functional potential of microbial communities

**FIGURE 6:**
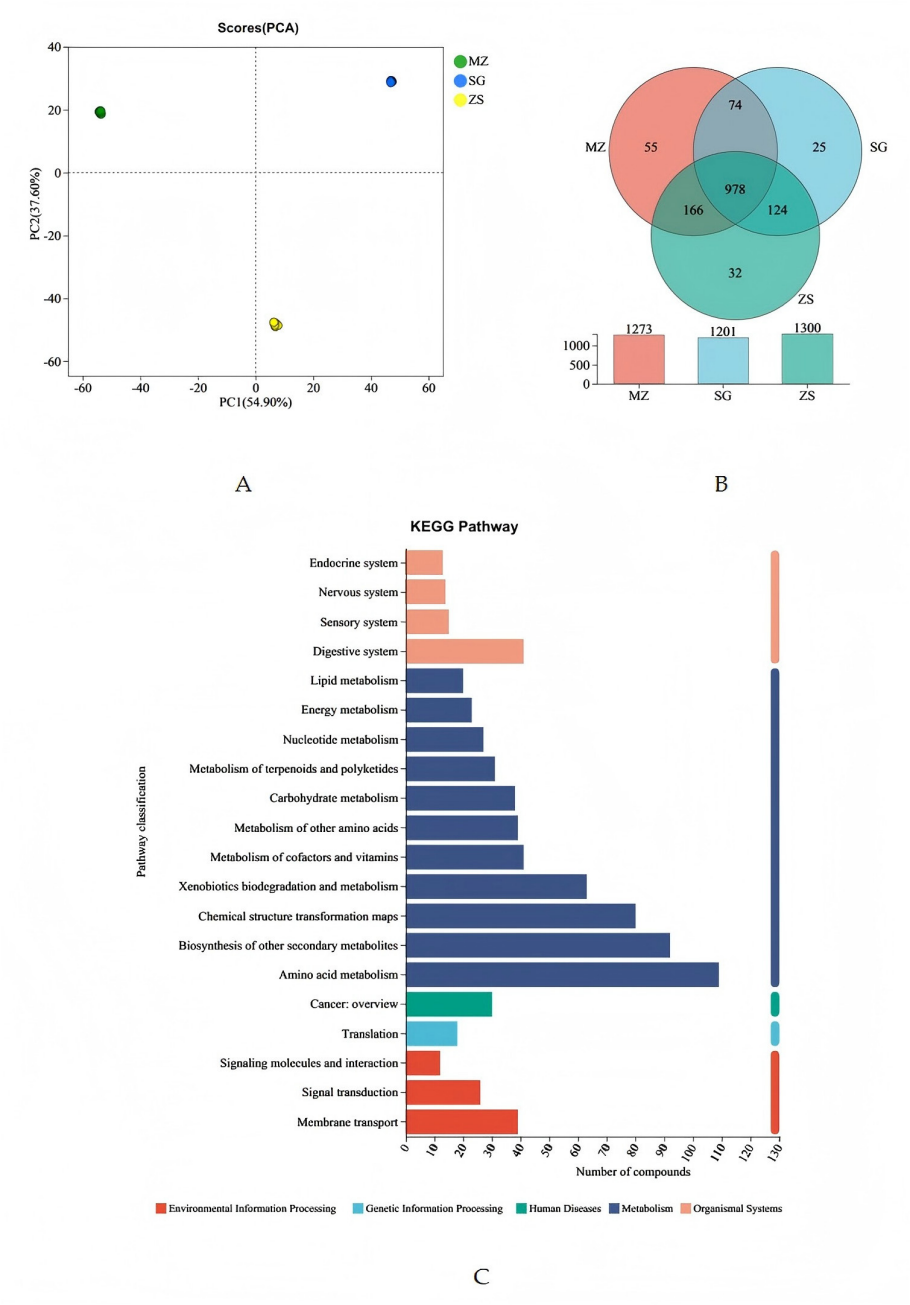
Analysis of the metabolite composition of fermented Chinese mustard greens from different regions of Guangdong Province, China. **(A)** Principal coordinates analysis. **(B)** Metabolite Venn diagram. **(C)** KEGG pathway classification: metabolites detected and annotated. MZ, SG, and ZS represent Meizhou City, Shaoguan City, and Zhongshan City, respectively.

The prediction of PICRUSt2 function was employed to forecast the functional information of microbial communities of FCMG and further understand the potential microbial functional characteristics. As shown in Figure 6, the dominant functions of bacteria in the MZ, SG, and ZS samples were almost identical, mainly involving metabolism (Supplementary Figure S1A). Among them, the abundance of carbohydrate metabolism and amino acid metabolism was higher (Supplementary Figure S1B). Combined with further KEGG data information (third level), we confirmed correlations between bacterial communities and pathways related to secondary metabolite biosynthesis, microbial metabolism in various conditions, and amino acid biosynthesis in FCMG from the three regions (Supplementary Figure S1C).

### 3.2 Fermented Chinese mustard greens metabolites analysis

#### 3.2.1 Metabolite components of fermented Chinese mustard greens

PCA was performed to preliminarily assess overall metabolic variations among groups and the variation within each group. As shown in Figure 6A, the contribution rate of the 1st principal component was 54.9%, the contribution rate of the 2nd principal component was 37.6%, and the total contribution rate was 91.5%, indicating that the model can correctly identify all samples of FCMG from the diverse areas.

The Venn diagram further illustrates the similarities and differences of metabolites in FCMG from the MZ, SG, and ZS regions. As shown in Figure 6B, the MZ, SG, and ZS samples contained 1,273, 1,201, and 1,300 named metabolites, respectively. Among them, 978 metabolites were present in FCMG in all three regions, mainly consisting of amino acids such as arginine, leucine, glycine, valine, proline, histidine, and aspartic acid. In addition, there were unique metabolites present in FCMG from the three areas, with the MZ, SG, and ZS samples containing 55, 25, and 32 unique metabolites, respectively. These unique metabolites may be one of the factors contributing to the different flavors and nutritional components of FCMG in the three areas.

The KEGG was utilized to annotate all the identified metabolites, statistically analyze the annotation status of the level 2 pathways. As shown in Figure 6C, under the secondary categorization of the KEGG metabolic pathways, the number of compounds annotated to metabolic pathways was the highest. The most abundant metabolism was amino acid metabolism, with 109 compounds, followed by biosynthesis of other secondary metabolites, chemical structure transformation maps, xenobiotics biodegradation and metabolism, and the metabolism of cofactors and vitamins with 92, 80, 63, and 41 compounds, respectively.

#### 3.2.2 Screening and identification of differential metabolites

**FIGURE 7:**
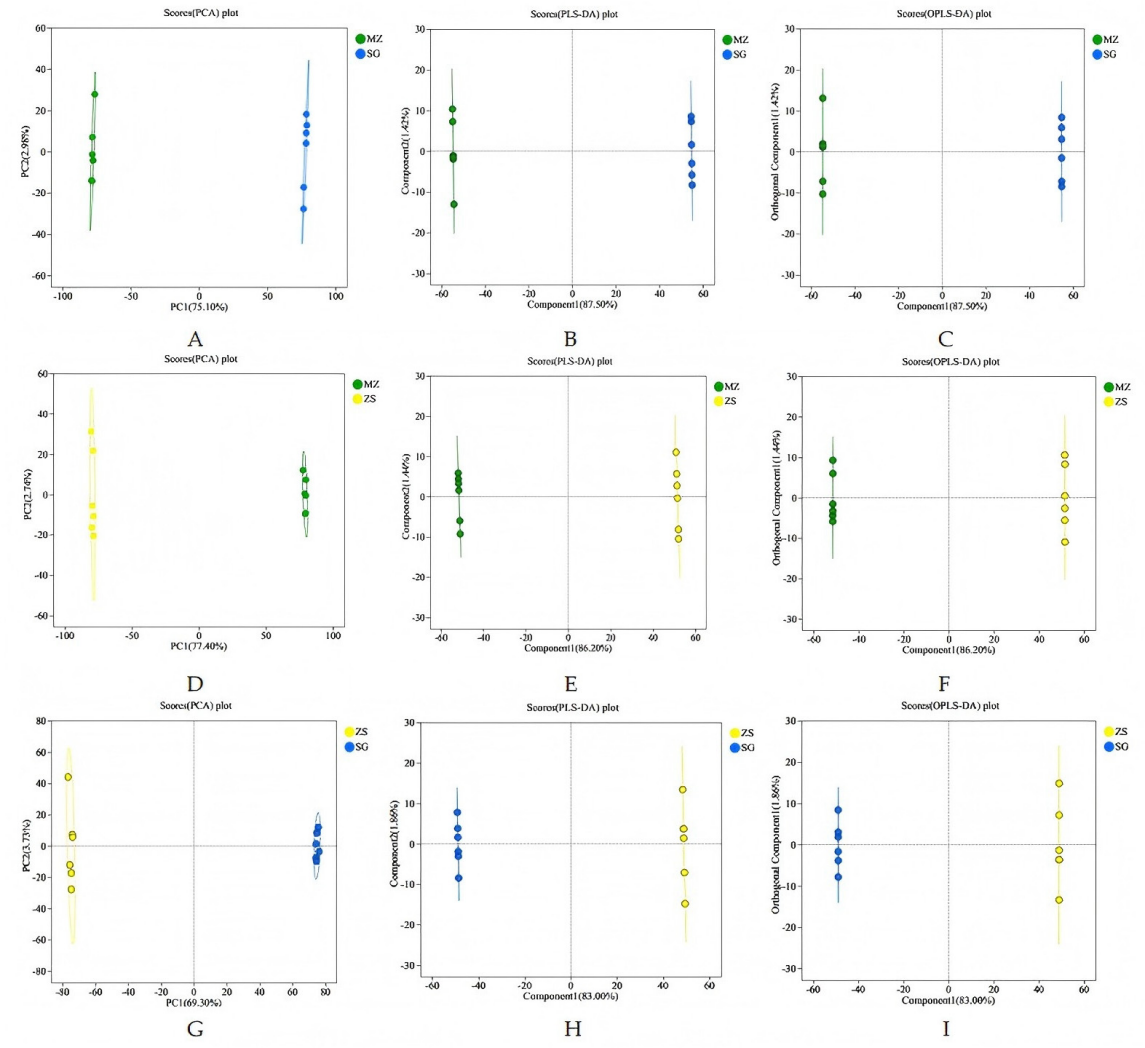
Volcanic diagram of the differential metabolites of fermented Chinese mustard greens from three areas of Guangdong Province, China. **(A)** MZ vs. SG; **(B)** MZ vs. ZS; **(C)** ZS vs. SG. MZ, SG, and ZS represent Meizhou City, Shaoguan City, and Zhongshan City, respectively.

**FIGURE 8:**
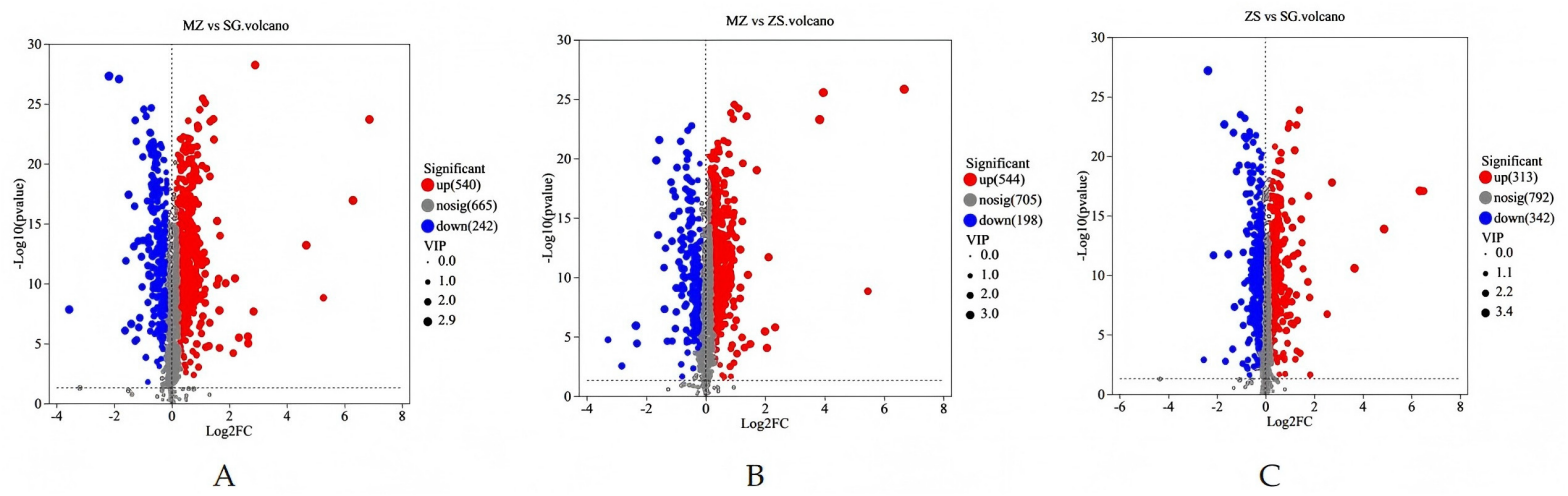
PCA, PLS-DA and OPLS-DA analysis of fermented Chinese mustard greens from three areas of Guangdong Province, China. **(A–C)** MZ vs. SG; **(D–F)** MZ vs. ZS; **(G–I)** ZS vs. SG. MZ, SG, and ZS represent Meizhou City, Shaoguan City, and Zhongshan City, respectively.

The FCMG from different regions were compared pairwise, and the PCA, PLS—DA, and OPLS—DA of different comparison groups are shown in Figure 7. The metabolic profiles of MZ and SG (Figures 8A–C), MZ and ZS (Figures 8D–F), and ZS and SG (Figures 8G–I) showed significant changes between the different groups.

In the volcano plot, red dots denote significantly elevated metabolites, blue dots denote significantly suppressed metabolites, and gray dots denote non-significant metabolites. According to Figure 7, MZ had 540 upregulated and 242 downregulated metabolites compared to SG (Figure 7A). Compared with ZS, MZ had 544 upregulated and 198 downregulated metabolites (Figure 7B), and compared with SG, ZS had 313 upregulated and 342 downregulated metabolites (Figure 7C). More detailed metabolite changes can be found in Supplementary Tables 2–4. These metabolites with significant variations and stable overexpression are likely to participate in important metabolic pathways and exercise important biological functions.

#### 3.2.3 Advanced pathway analysis of differential metabolites

**FIGURE 9:**
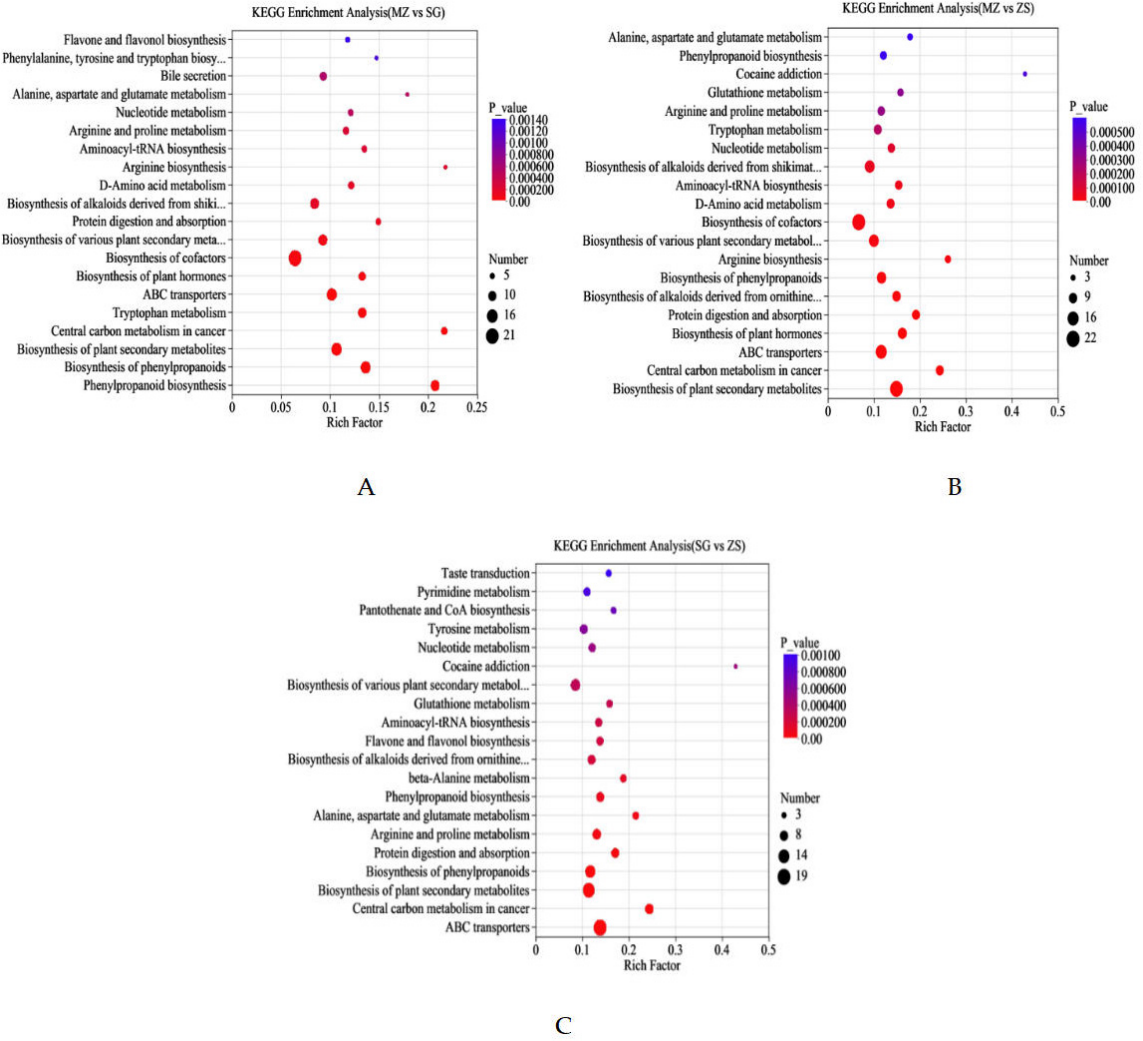
Advanced analysis of differential metabolites in fermented Chinese mustard greens from three areas of Guangdong Province, China, with an enriched bubble map illustrating metabolic pathway impact factors: **(A)** MZ vs. SG; **(B)** MZ vs. ZS; **(C)** ZS vs. SG. MZ, SG, and ZS represent Meizhou City, Shaoguan City, and Zhongshan City, respectively.

**FIGURE 10:**
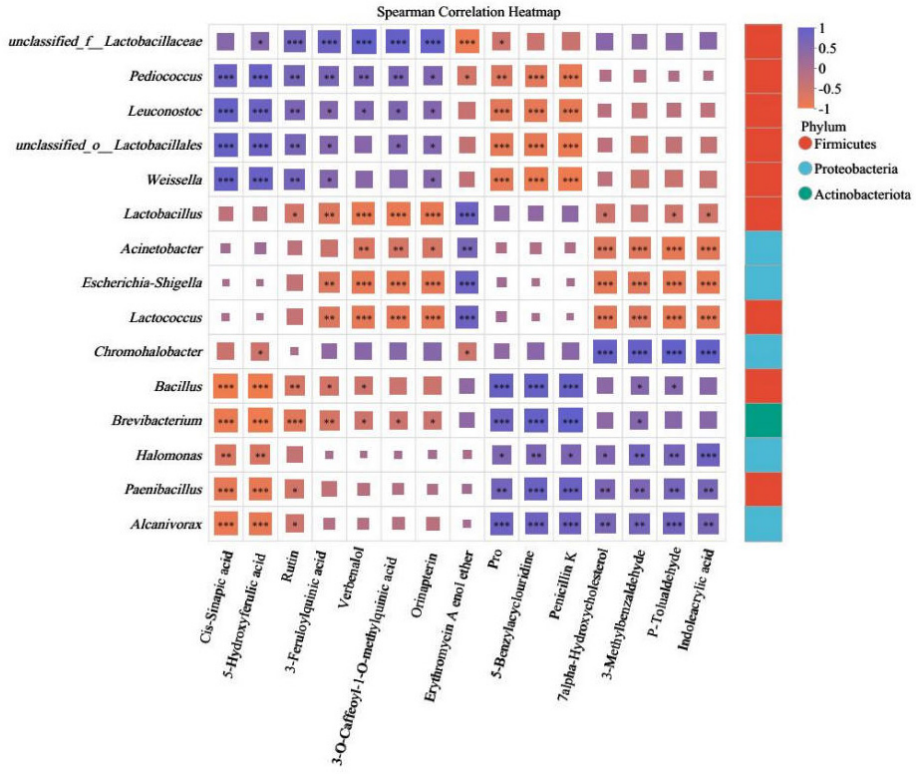
Spearman correlation analysis of metabolites and microbial communities among fermented Chinese mustard greens. The Spearman correlation coefficient r ranges from - 1 to 1; *r* < 0 indicates a negative correlation, and *r* > 0 indicates a positive correlation. *, **, and *** represent significant correlations at 0.05, 0.01, and 0.001, respectively.

Differential metabolite pathway analysis effectively elucidates the metabolites’ functional impacts on biological pathways. KEGG metabolic analyses comparing the MZ vs. SG, MZ vs. ZS, and SG vs. ZS groups are shown in Figure 9, where the horizontal axis indicates enrichment rate and the vertical axis denotes KEGG pathways. Bubble size reflects the number of enriched metabolites, while bubble color indicates the significance of enrichment *p*-values. FCMG from the three regions were significantly enriched in pathways including cofactor biosynthesis, ABC transporters, biosynthesis of plant secondary metabolites, and phenylpropanoid biosynthesis. It is worth noting that further comparison of KEGG pathway enrichment between different regions (MZ, SG, ZS) can reveal the regional specific changes of metabolites at the pathway level in more detail. As shown in Figure 10, the metabolic pathways enriched in different comparison groups are different. In the comparison between MZ and SG, flavor and flavor biosynthesis and phyllalanine, tyrosine and tryptophan biosynthesis showed significant differences (Figure 9A). There were significant differences between MZ and ZS in alanine, aspartate and glutamate metabolism and phylpropanoid biosynthesis (Figure 9B); However, there were significant differences between SG and ZS in taste transmission and pyromidine metabolism (Figure 9C). These differences are not only reflected in the types of pathways, but also in the amount of enriched metabolites, indicating that the metabolic functions of FCMG in different regions have their own metabolic characteristics shaped by their respective environments on the basis of commonness.

### 3.3 Relationship between the microbial communities and metabolites

Understanding the correlation between microbial communities and metabolite production is crucial for assessing the quality of FCMG. Spearman’s rank correlation analysis was performed between the top 15 non-targeted metabolites and all microbial genera. Figure 10 displays the associations between metabolites and the 15 dominant genera. *Lactobacillaceae* was significantly positively related to Cis-Sinapic acid (*r* = 0.90), 5-Hydroxyferulic acid (*r* = 0.84), 3-Feruloylquinic acid (*r* = 0.82), and 3-O-Caffeoyl-1-O-methylquinic acid (*r* = 0.90), indicating *Lactobacillaceae*, as the core fermentation bacteria, drive the accumulation of organic acids. *Halomonas, Paenibacillus*, and *Alcanivorax* were significantly positively related to 7 alpha-Hydroxycholesterol (*r* = 0.54, 0.59, 0.64), 3-Methylbenzaldehyde (*r* = 0.70, 0.61, 0.68), and P-Tolualdehyde (*r* = 0.66, 0.67, 0.72), indicating they may be involved in the metabolism of volatile substances and the synthesis of flavor compounds, such as alcohols and aldehydes. In addition, there was a high correlation between Lactobacillaceae and Rutin (*r* = 0.71). Rutin is a flavonoid compound widely present in plants, with antioxidant, anti-inflammatory, and other activities (Forouzanfar et al., 2025). There are research reports that certain lactobacilli secrete β-glucosidase or α-rhamnosidase, which can cleave the glycosidic bond of Rutin, release quercetin, and enhance its activity (Ding, 2019).

The independent analysis of three regions shows that there is significant regional specificity in the association network between microorganisms and metabolites, which directly affects the quality of products. In the MZ region, there is a significant positive correlation (*r* = 0.81) between *Lactobacillus* and Penicillin K; The strong negative correlation (*r* = 0.93) was observed between *Bacillus* and Penicillin K. Meanwhile, the unique *Achromobacter* species in the region showed a significant positive correlation (*r* = 0.94) with 7 alpha Hydroxycholesterol, suggesting that it may dominate the hydroxylation metabolic pathway of cholesterol, which is a key step in the formation of specific flavor precursors (Supplementary Figure S2A). In the SG region, the dominant bacterial group *Lactobacillus* is significantly positively correlated with 5-hydroxyferulic acid (*r* = 0.88), which is consistent with the overall analysis (Supplementary Figure S2B). The dominant strain in the ZS region is mainly *Lactobacillus*, which is significantly negatively correlated with Rutin (*r* = 0.83), while *Enterococcus* (*r* = 0.94), *Escherichia Shigella* (*r* = 1.00), and Rutin are significantly negatively correlated and positively correlated. This indicates that in the specific fermentation environment of ZS region, different microbial genera may play completely opposite roles (Supplementary Figure S2C). Some microbial communities degrade Rutin, while the presence of other microbial communities may be related to the synthesis of Rutin. Together, they form a complex metabolic balance network, ultimately determining the final composition of functional active ingredients in the product.

## 4 Discussion

The microbial community has a crucial impact on the quality, flavor, safety, and nutritional value of fermented vegetables. The metabolic activities of different microorganisms can dominate the fermentation process, affecting the final characteristics of the product (Li et al., 2023). In this study, *Lactobacillus* is the dominant strain in FCMG from MZ, SG, and ZS. This is also similar to the dominant bacterial strains found in other fermented vegetables in China and other countries (Rhee et al., 2011; Vittorio et al., 2012), such as suan cai (Yang et al., 2014), kimchi (Hong et al., 1994; Mah et al., 2004), khalpi (Tamang et al., 2005), sour bamboo shoot (Chen et al., 2022) and chili pepper (Xiao et al., 2023). Although *Lactobacillus* generally is dominant bacterial in MZ, SG, and ZS samples, consistent with that in other fermented vegetables worldwide, there are regional differences in sub dominant strains. The relative abundance differences of microorganisms such as *Bacillus, Leuconostoc, Pediococcus, Chromohalobacter, Paenibacillus, Weissella, Brevibacterium, Escherichia-Shigella, and Alcanivorax* are significant. The longer fermentation times of MZ (40 days) provides a more favorable environment for the growth of acid-resistant *Bacillus*. The shorter fermentation times of SG (15 days) can preserve a wider range of microorganisms, including *Leuconostoc* and *Pediococcus*, allowing it to adapt to high concentration salt environments. These differences can be attributed to a combination of geographical factors and variations in the natural fermentation process, which is influenced by regional location, fermentation time, salt concentration, and traditional brine composition (Yongsawas et al., 2023).

Based on the LC-MS untargeted metabolomics analysis, a total of 978 identical metabolites were identified in FCMG from the three regions (MZ, SG, and ZS), mainly consisting of amino acids such as arginine, leucine, glycine, valine, proline, histidine, and aspartic acid. These amino acid metabolites play multiple key roles in fermented vegetables, directly affecting their flavor, nutrition, shelf life, and health value (Liu et al., 2025; Yang L. et al., 2024). Amino acids can generate volatile substances through decarboxylation, deamination, and other reactions, giving fermented substances a special odor (Lee et al., 2011). The metabolism of aspartic acid and glutamic acid can produce organic acids, balancing the excessive sour taste of fermented vegetables. Amino acids can also react with reducing sugars to produce melanoidins, giving fermented vegetables a beautiful color (Purwandari et al., 2025). In addition, certain amino acid metabolites can also suppress the growth of spoilage bacterial and prolong the shelf life of fermented vegetables (Wang et al., 2025). We annotated all the identified metabolites using the KEGG database to evaluate their impact on biological metabolic processes. Under the secondary classification of KEGG pathways, the metabolism with the most annotated compounds was amino acid metabolism, similar to the microbial function prediction results. The predicted microbial pathway matched the actual metabolites, indicating that the genomic potential of the microorganisms was expressed in actual fermentation. In addition, this may also indicate that the changes in amino acid metabolism in fermented vegetables may be directly driven by the metabolic activities of microorganisms (Tamang et al., 2016).

This study systematically revealed the relationship between microbial communities and metabolites during the fermentation process through correlation analysis (Kumar and Parikh, 2025). Overall, as the core fermentation bacterium, *Lactobacillaceae* is significantly positively correlated with various organic acids, confirming its key role in driving the accumulation of acidity in fermented mustard (Zhang et al., 2025). The association between bacterial genera such as *Halomonas* and *Paenibacillus* and volatile substances suggests that these microorganisms may play important roles in the synthesis and metabolism of flavor compounds, collectively constituting the basic flavor of the product. It is particularly worth discussing that the dominant strain of *Lactobacillus* in ZS region is significantly negatively correlated with rutin, while *Enterococcus* and *Escherichia-Shigella* are significantly positively correlated with rutin (Fiorella et al., 2015). This indicates that different microorganisms may play completely opposite roles in specific fermentation environments. Some microbial communities degrade rutin, while other microorganisms may be involved in the synthesis of rutin. They together form a complex metabolic balance network, ultimately determining the final composition of functional active ingredients in the product. In summary, the quality of FCMG is not determined by a single microbial community, but by a complex microbial community that dynamically regulates metabolic pathways through collaboration, competition, and division of labor under specific environmental conditions.

## 5 Conclusion

This study comprehensively revealed the relationship between microbial communities and metabolome profiles of traditional FCMG from different regions (MZ, SG, and ZS) in Guangdong province. *Lactobacillus* is the dominant genus from all regions, yet significant variations microbial communities and metabolite composition existed in different regions. This study clarified the core role of *Lactobacillaceae* in accumulating various organic acids and rutin. It also revealed the specific associations of *Halomonas* and *Paenibacillus* with volatile flavor compounds. These new findings provide scientific basis for screening benefit microbial and develop flavor-oriented FCMG product. The good quality flavor FCMG can be achieved through regulating the proportions of the dominant functional microorganisms. In the future, the specific metabolic functions of these dominant strains should be verified through *in vitro* cultivation and conduct fermentation research based on these findings, thereby enhancing product quality and safety, and ultimately advancing the commercial production of FCMG.

## Data Availability

The data presented in the study are deposited in the NCBI repository, accession number PRJNA1428310.

## Footnotes

1
http://www.hmdb.ca/

2
https://metlin.scripps.edu/

3
https://cloud.majorbio.com

4
http://www.genome.jp/kegg/
