# Supplementary material for: Microbial communities and metabolome profiles of fermented Chinese mustard greens from diverse regions in Guangdong province, China
Source: Front Microbiol. 2026 Mar 17;17:1764488. doi: 10.3389/fmicb.2026.1764488 (PMC13036155; doi:10.3389/fmicb.2026.1764488)
Supplement: Supplementary file 1 [file Table_1.docx]

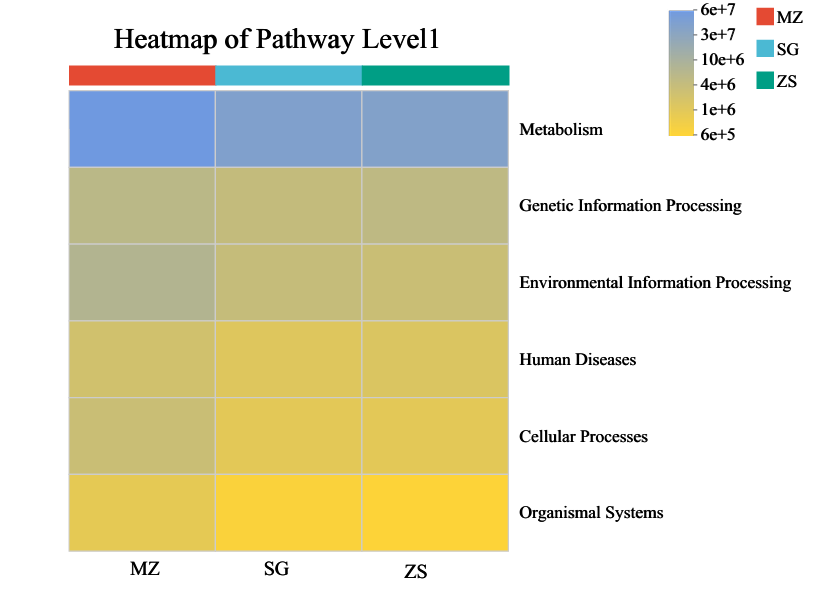

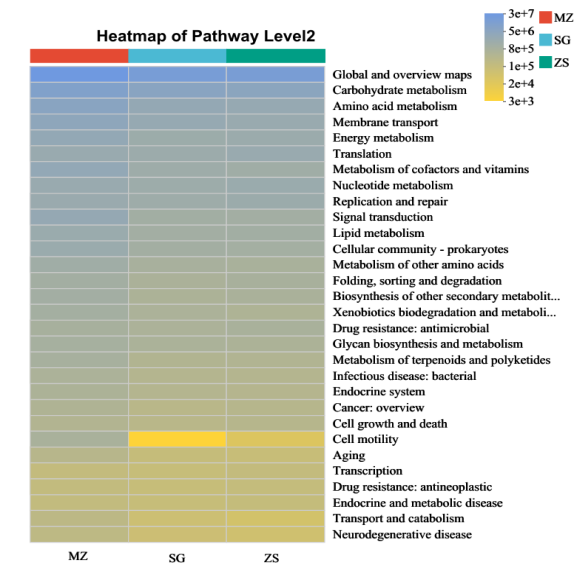


A B


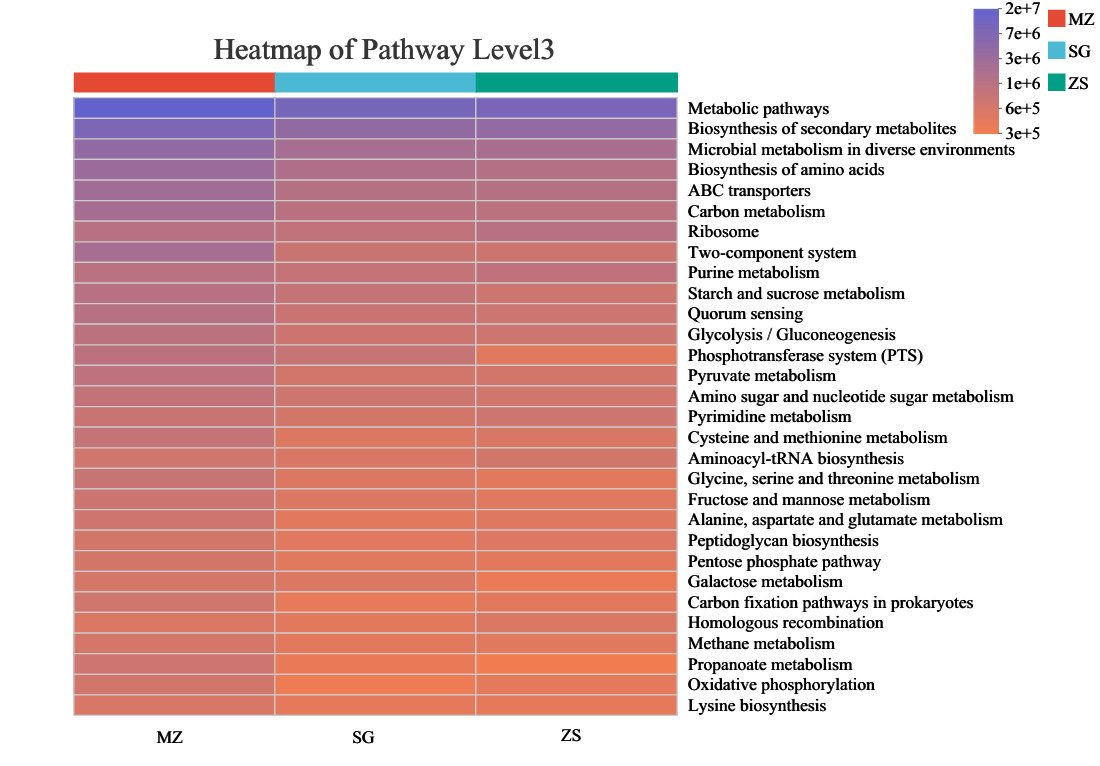


C

**FigS 1**

The bacterial microbiota's functional prediction using PICRUSt2 and the KEGG database, showing KEGG pathway results at Levels 1 (A), Levels 2 (B), and Levels 3 (C). MZ, SG, and ZS represent Meizhou City, Shaoguan City, and Zhongshan City, respectively.


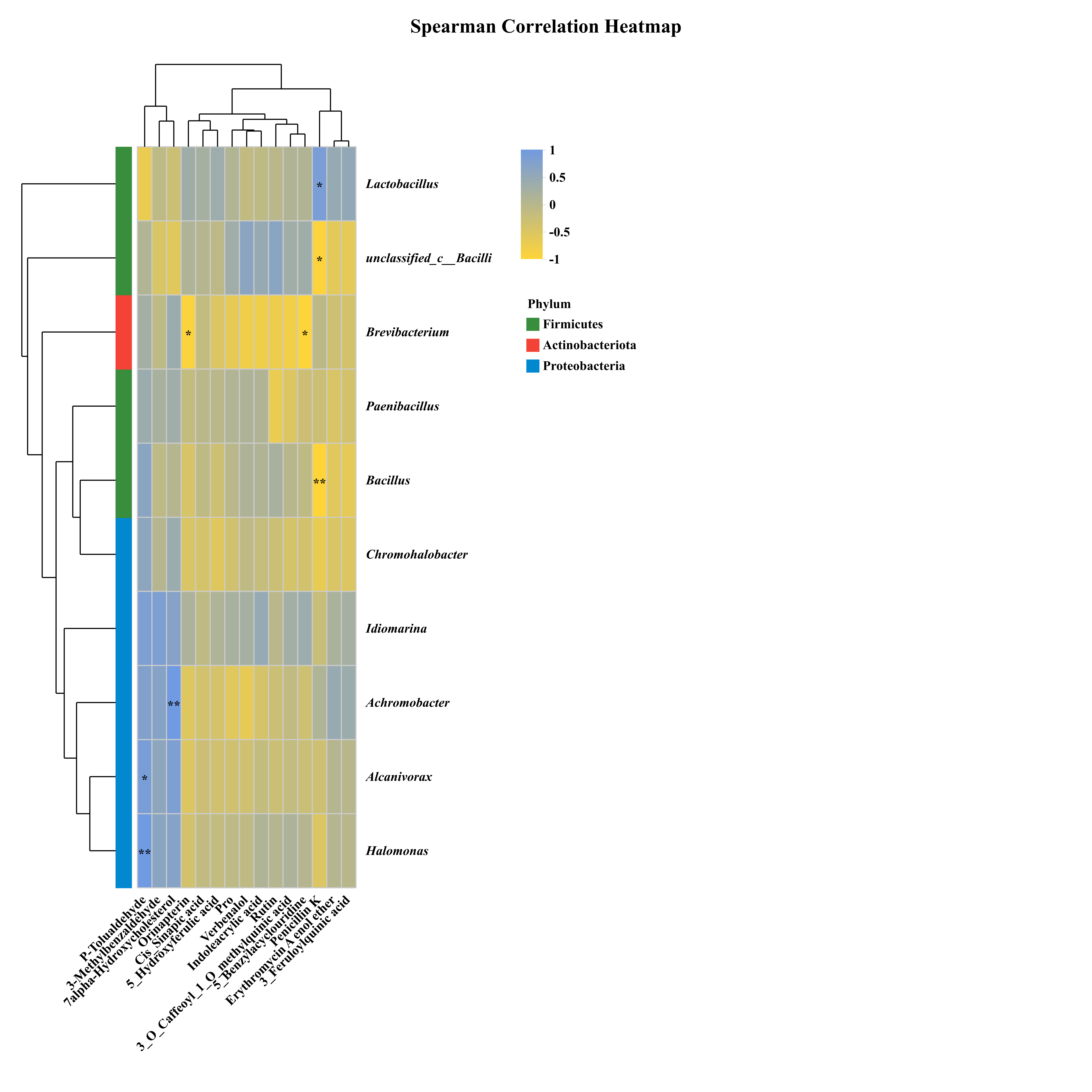

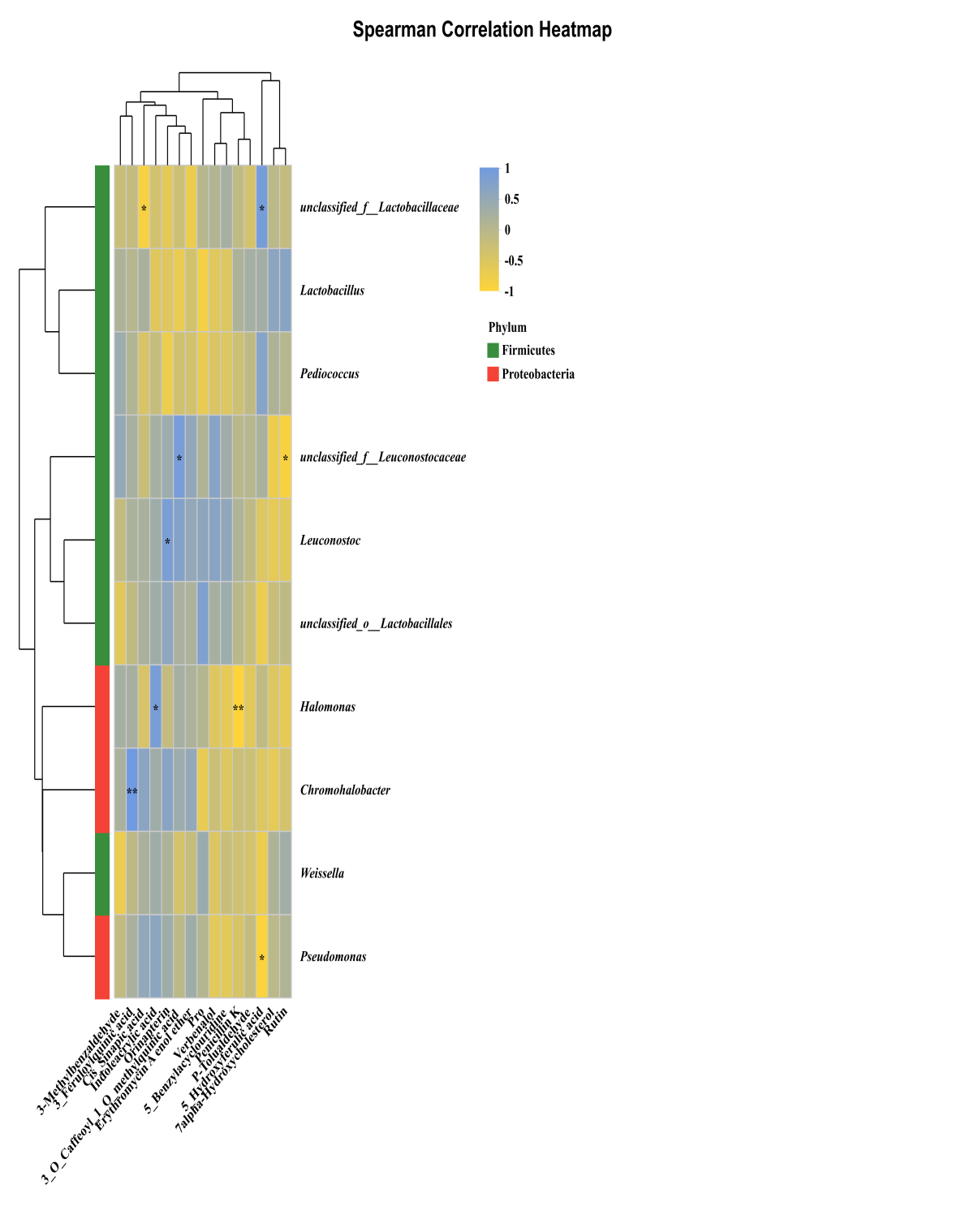


A B

**
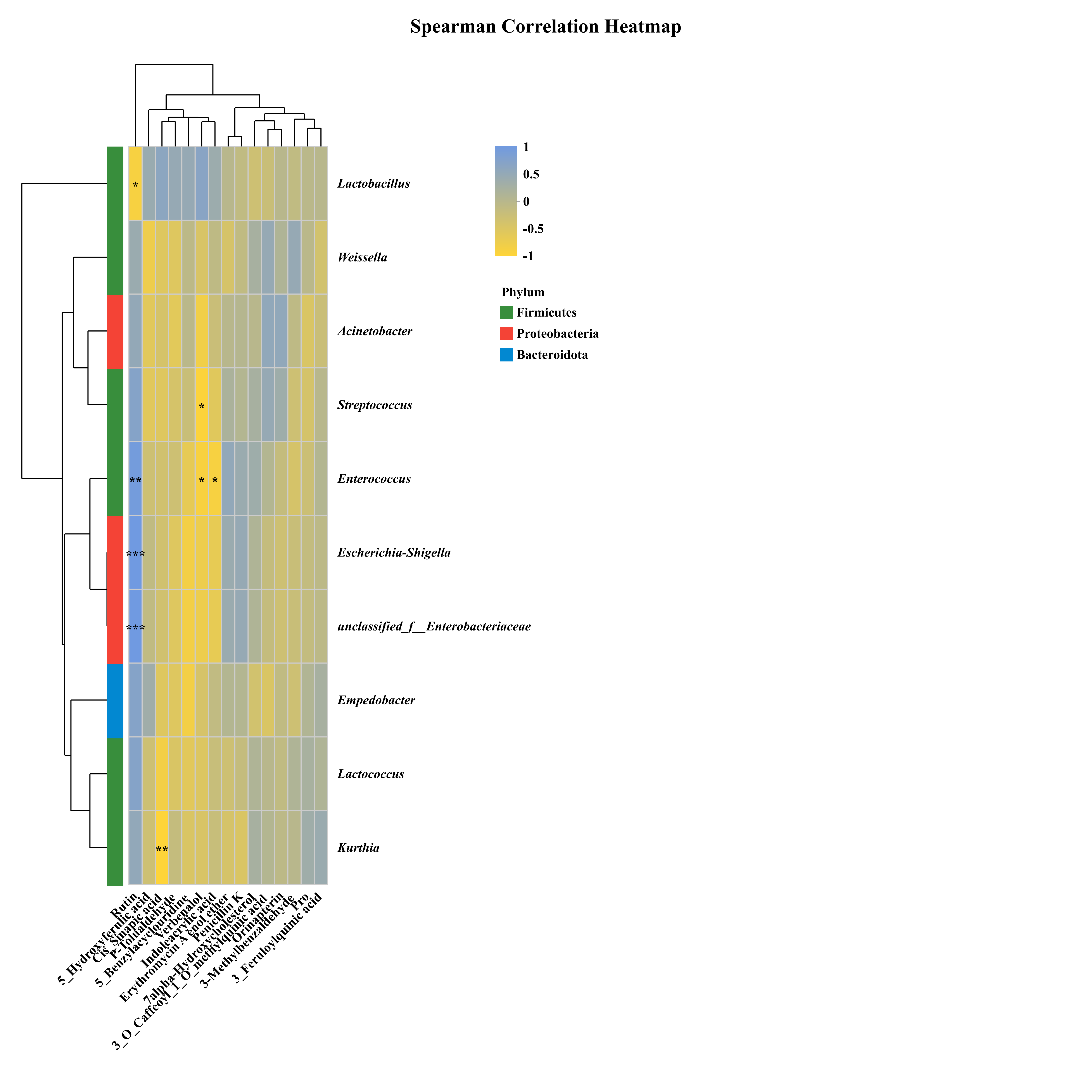
**

**C**

**FigS 2**

Spearman correlation analysis of metabolites and microbial communities among fermented Chinese mustard greens. (A):MZ; (B): SG; (C): ZS. The Spearman correlation coefficient r ranges from −1 to 1; r < 0 indicates a negative correlation, and r > 0 indicates a positive correlation. *, **, and*** represent significant correlations at 0.05, 0.01, and 0.001, respectively. MZ, SG, and ZS represent Meizhou City, Shaoguan City, and Zhongshan City, respectively.
